# Ethical implications of defining longstanding anorexia nervosa

**DOI:** 10.1186/s40337-024-01040-w

**Published:** 2024-06-11

**Authors:** Marthe M. Voswinkel, Simone M. Hanegraaff, Suzanne H.W. Mares, Elke Wezenberg, Johannes J.M. van Delden, Annemarie A. van Elburg

**Affiliations:** 1 Department of Eating Disorders (Amarum), GGNet Mental Health, Vordenseweg 12, Warnsveld, 7231 PA The Netherlands; 2 Department of Clinical Psychology, Utrecht University, Heidelberglaan 1, Utrecht, 3584 CS The Netherlands; 3 Bioethics and Health Humanities, Julius Center for Health Sciences and Primary Care, University Medical Center Utrecht, Universiteitsweg 100, Utrecht, 3584 CG The Netherlands

**Keywords:** Anorexia nervosa, SE-AN, Ethics, Lived experience perspective, Epistemic injustice

## Abstract

The label severe and enduring anorexia nervosa (SE-AN) is widely used in the literature on longstanding anorexia nervosa (AN). However, the process of constructing the criteria and the use of the label SE-AN has ethical implications that have not been taken into account. Through combining existing literature and lived experience perspective, this paper addresses to what extent the current criteria do and do not reflect the lived experience. Arguments are presented on why the process of constructing the criteria for SE-AN and the application of the label can be both identified as, and give rise to, epistemic injustice. Epistemic injustice is an injustice that is done to a person as an individual with the capacity of acquiring and sharing knowledge. This type of injustice can occur at any stage of an interaction between people in which knowledge is shared with one another. The paper concludes by giving suggestions on how to pursue epistemic justice in the process of defining longstanding AN.

## Abstract (plain language summary)

Some patients with anorexia nervosa (AN) develop a longstanding eating disorder. Over the years different labels, such as ‘chronic AN’ or ‘treatment resistant AN’, have been used to describe longstanding AN. Currently, the label that is used most often in scientific literature is ‘severe and enduring AN’ (SE-AN) and criteria for what entails SE-AN have been proposed. This paper looks at the possible ethical issues that have arisen in the process of constructing the criteria and possible ethical problems that may result from using the label SE-AN. The paper focuses on injustice that may arise when people with specific experiences, such as lived experience of longstanding AN, are not acknowledged as people with important knowledge to share. The paper concludes by giving suggestions on how to overcome this type of injustice.

## Introduction

Anorexia nervosa (AN) is a complex disorder that may in some patients evolve into an enduring illness. Although findings are highly variable, available studies have suggested that AN may progress to a long-lasting disorder in a sizable proportion of individuals [1–4]. Over the past decades, the group of patients that develop a persistent form of AN has been assigned different labels. From the 1980’s onwards, the term ‘chronic’ AN emerges in research papers on AN, differentiating chronic AN from a more acute form of the disorder [5]. Other examples of labels that have been used are ‘treatment-resistant’ and ‘refractory’ AN [6]. For years, ‘chronic AN’ was the most common label. While the term chronic implies that one will not recover from the disorder, this is not necessarily the case, as empirical studies with long follow-ups have shown that even those who have suffered from AN for over a decade are still able to reach full recovery [7]. The label ‘chronic’ thus runs the risk of leaving patients without hope for the future, even when hope might still be warranted.

In more recent years, we have seen an increase in the use of the label ‘severe and enduring AN’ or SE-AN. This label is believed to have less negative implications, and SE-AN has now been widely adopted as an alternative to ‘chronic’(6). In this paper, longstanding AN will be used to refer to a persisting form of AN. Although the SE-AN label is now widely used, consensus on the precise definition of SE-AN and its criteria has not been reached. In an attempt to create well-defined criteria, Hay and Touyz have proposed three criteria: 1) “clinically significant functional impact i.e. impoverished and poor quality of life, with unrelenting symptoms”, 2) “duration of several years (minimum three) of AN” and 3) “exposure to at least two evidence based treatments” [8]. However, the empirical evidence supporting these criteria is still limited. Identifying what characterizes patients with longstanding AN has proven to be complex. The fact that no consensus has been reached on the definition and its criteria is problematic, since without consensus on a definition, study populations diverge which reduces the overall generalizability of study results. Furthermore, there are serious ethical concerns that need to be considered during the process of defining and labelling longstanding AN. In this paper, we will focus specifically on the ethical concerns with regard to sharing and creating knowledge. To do so, we will use the framework of epistemic injustice. The paper will start by explaining the concept of epistemic injustice. Following, existing literature will be combined with views from both clinical and lived experience with longstanding AN in order to show that the current criteria may not completely capture what longstanding AN entails. Next, the paper will present why both the process of constructing and using the label and its criteria could be considered an instance of epistemic injustice. We will conclude by giving suggestions on how to pursue epistemic justice in defining longstanding AN.

## Epistemic injustice

One of the distinctive features of human beings is the ability to think, to reason and to know. As humans, we use different sources to acquire knowledge, such as perception and memory, yet a great deal of what we know stems from our interaction with other people [9]. We use knowledge—in the broadest sense of the word—to make sense of our experiences and the world we live in. Any individual with the ability to acquire and share knowledge, can be referred to as a ‘knower’. In epistemic injustice, a concept first proposed by Miranda Fricker in 2007 [10, 11], this ability is affected. Fricker defines epistemic injustice as “a wrong done to someone specifically in their capacity as a knower” [10]. In other words, epistemic injustice arises when an individual is harmed in their capacity of acquiring or sharing knowledge. This can, for instance, happen when an individual is not acknowledged as someone with knowledge to share, either by not being taken seriously or by not being given the opportunity to share knowledge in the first place. Epistemic injustice is linked to social identities and the prejudice that exists around these identities. Some social identities are seen as more trustworthy than others. For instance, healthcare professionals are often believed to be trustworthy, whereas those with mental illness, such as AN, may be seen as less trustworthy. One of the central harms of epistemic injustice is that, due to this prejudice, a person is not acknowledged as a knower. In severe cases this may lead to people being approached as objects to have knowledge about.

Within the general concept of epistemic injustice, Fricker distinguishes two main types relating to “two of our most basic everyday epistemic practices: conveying knowledge to others by telling them, and making sense of our own social experiences” [10]. In the current paper, we will concentrate mainly on the injustice that arises in the process of conveying knowledge to others and its consequences. When conveying knowledge to others, a person may not be taken seriously or believed or may even be excluded from sharing knowledge as a result of existing prejudice. This is what Fricker calls “testimonial injustice” [10]. ‘Testimonial’ refers to the process of giving testimony, often associated with legal cases, yet as a general term, testimony refers to any situation in which knowledge is conveyed from one person to another [9], hence ‘testimonial injustice’. Using an example within the context of eating disorders, testimonial injustice may arise when, due to the stereotypical image of someone with AN, what is said is misinterpreted as originating from the eating disorder and therefore disregarded [12]. When relevant information is disregarded as a result of prejudice, or when people with a specific social identity, such as patients with an eating disorder, are not given enough opportunity to share their social experiences, the collective knowledge pool remains incomplete and may be one-sided. As a consequence, this may lead to the development of concepts, labels or criteria that may not necessarily be representative of the lived experience.

## The SE-AN label, criteria and lived experience

If we were to use the criteria as they are proposed by Hay and Touyz, a patient with SE-AN would have AN with unrelenting symptoms—such as being underweight as a result of restricted food intake, a distorted body image and preoccupation with weight—causing significant clinical impact for a minimum of three years for which there has been exposure to at least two evidence based treatments [8]. The question arises whether these criteria encompass the experience of people with lived experience of longstanding AN. Unfortunately, the number of studies conducted specifically on how patients with longstanding AN perceive the proposed criteria is small [13, 14] resulting in limited knowledge on the view of these patients. Some qualitative studies have focussed on the lived experience of patients with longstanding AN and thus provide insight into whether the criteria for SE-AN grasp the lived experience.

In a study by Broomfield, Rhodes and Touyz [13] people with lived experience were given the opportunity to share their perspectives on defining and labelling longstanding AN. Duration of illness as a criterion was suggested by about half of the participants. In contrast to the proposed minimum duration of three years by Hay and Touyz [8], people with lived experience, including one of the current authors, suggest a longer time frame. An illness duration of seven years is reported by some participants in the study by Broomfield et al. [13]. This corresponds with criteria used in an earlier randomized trial by Touyz et al. [15]. The fact that these timeframes vary between studies can be seen as a sign of the complexity of determining what characterizes people with longstanding AN.

Reay et al. [14] included both participants with lived experience and healthcare professionals to investigate what criteria were deemed appropriate in the definition of longstanding eating disorders. The continuous pattern of relapse and improvement is a characteristic of longstanding eating disorders that was mentioned by almost all participants in this study. This is recognized by the authors of the current paper from both clinical and lived experience. However, this specific aspect has not been included in the proposed criteria for SE-AN.

Concerning the label SE-AN from a lived experience perspective, literature showed that, while the use of any label at all was questioned, ‘enduring’ was more representative of their experience than ‘severe’ [13, 14]. One explanation given by a participant was the fact that physical severity could vary greatly from time to time. Another explanation could be that within the context of eating disorders severity is often expressed as low bodyweight, whereas studies showed that people with AN have strong doubts about weight as a criterion for longstanding AN, given that AN is a mental illness [13, 14, 16]. Moreover, using low bodyweight as a criterion could exclude patients with a normal weight that fulfil all other criteria for longstanding AN and suffer from irreparable damage to physical health due to having been severely underweight. In fact, these irreversible physical issues are even mentioned as a possible criterion for longstanding AN by people with lived experience [13].

All in all, based on both literature and clinical and lived experience of the current authors, there is ground for the tentative conclusion that the currently used label SE-AN and the proposed criteria for longstanding AN do not fully capture the lived experience.

## Defining and labelling SE-AN as epistemic injustice

In the following paragraphs, we will argue why the concept of epistemic injustice could be applicable to the process of constructing the SE-AN label, its criteria, and the application of the label. First, we will address the fact that people with longstanding AN have not sufficiently been acknowledged as knowers in the process and that this can be identified as an instance of epistemic injustice. Thereafter, we will discuss how the SE-AN label can give rise to testimonial injustice.

What becomes clear through examining the existing literature, is the fact that people with lived experience have had limited opportunity to share their views on how they believe longstanding AN should be defined and labelled. More importantly, their views have not yet been incorporated in the criteria. As stated earlier in this paper, not being acknowledged as a knower, is one of the central harms of epistemic injustice. It seems that, in the process of establishing criteria for SE-AN, people with longstanding AN have primarily been treated as objects to have knowledge about, as is the case in the quantitative studies that have tried to elucidate what defines SE-AN [16–19]. One could argue that treating people as objects in quantitative studies is justified given that the research is aimed at improving care for these patients. They are not merely treated as means, but as ends as well. Nonetheless, the question remains whether a definition of longstanding AN is complete without the phenomenological perspective. Fortunately, the lived experience perspective received attention through qualitative research. The previously mentioned studies by Broomfield et al. [13] and Reay et al. [14] are examples of studies in which people with lived experience have been treated as knowers. As Broomfield et al. [13] wrote: *“It was hoped that by providing these individuals the opportunity to contribute to this debate, the process would empower this often marginalised group.”* The inclusion of people with lived experience in defining longstanding AN is a positive development. Yet, only two studies on a label that has been used for over a decade, seems far from sufficient, especially when the importance of lived experience has been emphasized by researchers in the field [20]. Also, even when people with longstanding AN were included through qualitative research, the research questions were still determined by researchers, often without lived experience. As a result, even qualitative research does not entirely prevent the occurrence of epistemic injustice. It is therefore important to include people with lived experience from the beginning of research projects onwards [21].

In addition to the epistemic injustice that has occurred in the process of constructing the label, the SE-AN label could give rise to testimonial injustice, where conveying knowledge is restricted as a result of prejudice. For example, the word ‘enduring’ in the label could be interpreted as either retrospective, prospective, or both. When applied in a prospective sense, it may be associated with the term ‘chronic’ and therefore have negative connotations. This may cause loss of hope in the patient but also affect attitudes of healthcare professionals, as is illustrated by one participants’ story of her psychiatrist calling her a “career anorexic” when her AN was considered chronic [13]. This example is in line with research showing that healthcare is not free of stigma on AN [21–24]. When healthcare professionals could interpret ‘enduring’ as ‘a patient will not recover’, this could affect the attitude of a professional. Possibly, a wish to reattempt treatment may be considered pointless and lead to the patient not being taken seriously in her request, which is a case of testimonial injustice. Diagnostic overshadowing is another example of testimonial injustice that may arise in healthcare. This has been personally experienced by one of the current authors with lived experience. In diagnostic overshadowing, physical complaints are unjustly ascribed to mental disorders [25]. After years of being underweight and having had a restrictive diet, healthcare professionals could be more inclined to ascribe physical issues to the history of longstanding AN, without further diagnostic evaluation. Apart from the harm done to the patient as a knower, this may also induce further physical harm, when other causes are overlooked. These are two examples of how a label may affect treatment possibilities. The fear that a label could restrict treatment options is mentioned by participants in both the studies by Reay et al. and Broomfield et al. [13, 14]. Including testimonials from the lived experience and increasing awareness in professionals how labels and stereotypes may affect their judgement are essential steps in moving forward.

In conclusion, these arguments show that both the construction and application of the SE-AN label have potential ethical implications that should be taken into account. This does not necessarily mean we should not attempt to define criteria and a label for longstanding AN altogether, as a label may serve as acknowledgement, as an explanation, and having clear-cut criteria may improve research. It does however mean that in the process of defining criteria and constructing a label for longstanding AN, people with lived experience should get the opportunity to share their experiential knowledge.

## Towards epistemic justice in defining longstanding AN

Fortunately, the ethical issues mentioned in the previous paragraph can be resolved by co-research [26]. Within the context of the current paper, we interpret co-research as an active partnership between researchers, people with lived experience and possible other stakeholders, such as significant others of those with lived experience. In this paragraph, we will first turn to how co-research can ameliorate epistemic injustice in general. Subsequently, we will turn to some more specific points to take into account when conducting co-research with individuals with longstanding AN.

Co-research can contribute to diminishing epistemic injustice and promoting epistemic justice in various ways. Firstly, what characterizes co-research, is the fact that, specifically, knowledge generated through lived experience is recognized and utilized [26]. Secondly, in co-research, those with lived experience are involved in the entire research process, from start to end. As a result, they can exert influence on and collaborate in all parts of the project, e.g. defining research questions, prioritize problems relevant for those who are supposed to benefit from it and improve the dissemination of findings [25–30]. Thirdly, an important aspect of co-research is continuous reflection on the process and collaboration [30, 31]. With regard to preventing testimonial injustice specifically, it may be important to reflect on how prejudice may subconsciously affect the collaboration between researchers with and without lived experience. Many recommendations, guidelines, frameworks and principles on co-research are available (see: [31–35], albeit different terms, e.g. co-creation and co-production, are used interchangeably to describe a similar process [36]. However, to date, little has been written about co-research in eating disorder research specifically [37]. We therefore conclude this paper with some points of interest that may be of importance in co-research with people with longstanding AN based on our lived, clinical and research experiences.

In order to achieve epistemic justice with regard to defining longstanding AN, we could start by reassessing whether a label is necessary and if so, redefine the label and its criteria, via co-research with people with lived experience. In order to pursue epistemic justice, it would be recommended to include a diverse population of people with lived experience as their perspectives on longstanding AN may vary. To do so, approaching a broad range of recruitment sites could be fruitful, since some of the patients with longstanding AN receive care outside of eating disorder treatments centers. One could think of providers of alternative therapies, paramedical care or care for comorbid conditions such as personality disorders, autism, or physical complaints due to having AN. Furthermore, recruitment via mental health recovery groups, self-help organisations and providers of education of using experiential expertise in healthcare could be helpful. As with any research, it is important to consider what would motivate or discourage the intended contributors to get involved [38]. A perceived barrier could be, for instance, the fear to (partially) lose social benefits related to voluntary work, as some people with longstanding AN receive social benefits. Clear communication about this subject is necessary. Another example of a possible barrier to get involved in research, may be a reduction of, or great fluctuations in, capability. This makes differentiation and flexibility of participation opportunities essential [39]: it creates a more equal opportunity for all relevant patients and can consequently increase both the number and the diversity of contributors. Offering the choice between either membership of the research team or participation through an online panel, could be a way to take differences in capability into account.

We recognize that these ideas and suggestions are only a start and not a fully developed guideline, yet we hope to offer the reader some inspiration and stimulate readers in thinking about co-research with people with longstanding AN.

## Conclusions

People with longstanding AN have had very limited opportunity to share their experiential knowledge in the process of defining and labelling longstanding AN. This can be identified as an instance of epistemic injustice. A great deal remains to be done in order to create an epistemically just definition of longstanding AN. The proposed ideas with regard to co-research on longstanding AN are by no means exhaustive, as there are many more options that could be applicable to co-research with people with longstanding AN. Since many questions on longstanding AN remain, further research is still warranted. The current authors intend to conduct research in collaboration with individuals with lived experience on what characterizes longstanding AN, with regard to comorbidity and views on recovery. As stated in the introduction, consensus on the criteria for and definition of longstanding AN has not been reached. While some may say this is problematic, we could also perceive it as an opportunity to pursue epistemic justice.

## Data Availability

No datasets were generated or analysed during the current study.

## Abbreviations

AN: Anorexia nervosa
SE-AN: Severe enduring anorexia nervosa
